# The Diagnosis of Aneurism

**Published:** 1895-11-16

**Authors:** A. Pearce Gould

**Affiliations:** Senior Assistant Surgeon to the Middlesex Hospital.


### THE DIAGNOSIS OF ANEURISM.

By A. Pearce Gould, F.R.C.S., Senior Assistant Surgeon to the Middlesex Hospital.

We have now to consider how the different varieties of aneurism may be recognised. A tubular or fusiform aneurism is recognised by the outline of the tumour, and by its very quiet course, which, in external arteries, is generally unattended with symptoms. In internal arteries, especially the thoracic aorta, the presence of a large tumour—although it be a fusiform aneurism—is almost invariably the cause of more or less grave "pressure-signs," while the dilated and inelastic artery at the very centre of the circulation is a potent cause of embarrassment to the heart. In intra-thoracic aneurism a combination of fusiform and sacculated aneurism is often met with owing to advanced disease in one part of the wall of a generally dilated artery. This combination is of the highest importance. In these pouches or sacs the wall is much weaker than in the fusiform part of the sac, and therefore in them expansion of the sac takes place more rapidly, and rupture is prone to occur.

An external sacculated aneurism is recognised by its more or less globular outline, and usually also by the intensity of the symptoms (chiefly "pressure-signs") to which it gives rise. If the increase in size of the tumour is rapid the pulsation will be found to be superficial, and the probability is very great that the sac is a very thin one, not strengthened by the deposit and organisation of fibrin, and the danger of rupture of the sac is correspondingly great. Of quite other import is the condition of rapid growth of the tumour with lessened force and distinctness of the pulsation, and this point is a very important one to keep in mind. The pulsation of an aneurism depends not only upon the contraction of the left ventricle of the heart, but also upon the resistance of the sac. Accordingly, when the sac has a small defect in it and allows the blood within it to leak out into the surrounding tissues, the pulsation becomes less marked, while the tumour rapidly enlarges. If a larger rupture of the sac occurs, so that the effect of the resistance of the sac to the force of the heart is entirely lost, and the blood pressure is wholly expended in propelling the blood into the planes of cellular tissue around the aneurism, pulsation is lost altogether, but the tumour suddenly and very greatly grows. To put it briefly, rapid enlargement of an aneurismal tumour with increased pulsation in it signifies a complete but enlarging sac with little or no clot within it; rapid enlargement of an aneurismal tumour with lessened pulsation generally signifies a leaking aneurism, while sudden and great enlargement of the tumour with entire loss of pulsation signifies a ruptured aneurism. Another sign is also met with when the sac of an aneurism is incomplete—the outline of the tumour is obscured. So long as the sac is entire, and the blood and clot are entirely within it, the outline of the tumour is distinct and well defined. With leaking of blood into the tissues around the sac this clear outline is blurred, and when blood is poured out into the planes of cellular tissue in fuller quantity the clear outline of the tumour is wholly lost. The intensity of the pulsation in an aneurism, and the distinctness of the outline of the tumour, are very important indications of the integrity of the sac, and they are, therefore, very valuable guides to treatment.

In this connection I must mention another—fortunately rare—complication of aneurism, which to some extent simulates rupture of the sac. Under some conditions acute inflammation may be lighted up around the sac. This generally occurs in the case of large tumours imperfectly supported by the surrounding tissues, and in some cases has seemed to be set up by much handling of the tumour, or by sudden solidification of its liquid contents. Hence this complication has been most often met with in the axilla, at the root of the neck, or in the groin. The acute inflammation is accompanied by free inflammatory effusion, which causes increase in the size of the swelling and indistinctness of its outline. In these respects the case resembles one of yielding of the sac. But there are the other usual phenomena of acute inflammation—superficial œdema, bright redness of the skin, increased local heat, throbbing pain, and general pyrexia. When pus forms the swelling over the aneurism may fluctuate, and this may render the nature of the case still more clear. The peril attending this complication is very grave. In some very fortunate cases the artery leading to the aneurism has been sealed by a process of plastic arteritis, even when a huge slough of aneurism aud tissues around it has been separated. More often the artery is opened up by ulcerative arteritis, and blood is poured into the suppurating area around the sac and escapes through an internal wound. Sloughing of the sac always follows if suppuration occurs around it, for the tissue of the sac is nourished not by the blood within its cavity, but by that which reaches it on its exterior, and if these vessels are destroyed by suppurative inflammation, a fatal anæmia of the sac results.

Diminished pulsation also accompanies the cure of an aneurism; if the sac gradually becomes firmer and smaller, with less marked and less expansile pulsation, it indicates that the sac is being gradually filled up with laminated clot. When in such a case the pulsation has lost its expansile character altogether, and is only "heaving," it shows that the whole aneurism is solid, but that the artery from which it springs is not occluded. Cure only takes place when the artery as well as the aneurism is obliterated, and this is shown by the tumour becoming entirely pulseless, as well as by other changes in the arterial system, such as at first loss of pulse in the arteries beyond the aneurism and then appearance of palpable pulsation in the large anastomosing arteries. The cure of an aneurism more often occurs by a mere sudden coagulation of the blood in the sac and artery, or at any rate the final stage of the coagulation occurs suddenly, and the pulsation suddenly disappears, and then if all goes well the solid sac slowly shrinks.

The last variety of aneurism that we need especially consider is that in which there is a direct communication between an artery and a vein. This is attended with very marked and characteristic signs. The veins of the part both on the distal and proximal side of the communication are varicose and greatly enlarged, and they are the seat of a very intense thrill and of a loud bruit. The bruit is often peculiarly musical in tone, or comparable to the buzzing of a fly in a paper box; it may be so loud as to be audible not only to the patient, but even to bystanders. No other bruit is so intense as this. A varicose aneurism is distinguished from an aneurismal varix chiefly by the history of the case, by recognising the presence of a pulsating tumour other than that of the varix, and also in some cases by the presence, in addition to the very loud artero-venous murmur of the characteristic blowing aneurismal bruit.

